# Infection-Related Glomerulonephritis Due to Subacute Bacterial Endocarditis in the Aortic Valve: A Case Report

**DOI:** 10.7759/cureus.62670

**Published:** 2024-06-19

**Authors:** Sushrut Gupta, Pranjal Kashiv, Sunny Malde, Shubham Dubey, Kapil N Sejpal, Amit S Pasari, Manish Balwani

**Affiliations:** 1 Nephrology, Jawaharlal Nehru Medical College, Datta Meghe Institute of Higher Education and Research, Wardha, IND

**Keywords:** burkholderia cepacia, hemodialysis, antibiotics, kidney biopsy, endocarditis, glomerulonephritis

## Abstract

Infection-related glomerulonephritis (IRGN) is a rare but severe complication of bacterial infections, including subacute bacterial endocarditis (SBE). We present a case of a 15-year-old male with bilateral lower limb swelling, facial puffiness, frothy urine, and dyspnea. Laboratory investigations revealed abnormal kidney function tests and imaging studies confirmed infective endocarditis. Blood cultures isolated Burkholderia cepacia and methicillin-resistant coagulase-negative Staphylococcus. Kidney biopsy confirmed immune complex-mediated glomerulonephritis. The patient received multidisciplinary care, including respiratory support, hemodialysis, antibiotics, and blood transfusion. This case highlights the importance of recognizing and promptly managing IRGN secondary to SBE to prevent irreversible renal damage and systemic complications.

## Introduction

Infection-related glomerulonephritis (IRGN) is a rare but severe complication of bacterial infections, particularly those caused by Streptococcus species and Staphylococcus aureus. It is characterized by the deposition of immune complexes in the glomeruli, leading to inflammation, impaired renal function, and potentially irreversible kidney damage [1]. IRGN typically presents with hematuria, proteinuria, hypertension, and renal insufficiency, often in the setting of an active or recent bacterial infection [2]. Subacute bacterial endocarditis (SBE) is a form of infective endocarditis characterized by a slower onset and less severe symptoms than acute bacterial endocarditis. It is commonly caused by viridans group streptococci and coagulase-negative staphylococci [3]. The cardiac manifestations of SBE can vary widely and may include murmurs, embolic events, and valvular insufficiency [4].

The association between infective endocarditis and IRGN has been well documented in the literature. Endocarditis-associated glomerulonephritis is thought to occur due to the deposition of immune complexes formed in response to the infecting microorganism, which then becomes trapped in the glomerular basement membrane, leading to renal inflammation and injury [5]. Prompt diagnosis and treatment of underlying bacterial infection and renal complications are essential to prevent further renal damage and systemic sequelae. Management typically involves antimicrobial therapy targeted at the causative organism, supportive care to maintain renal function, and, in severe cases, renal replacement therapy [6]. Given the potential for significant morbidity and mortality associated with IRGN secondary to SBE, clinicians must maintain a high index of suspicion and promptly evaluate and manage patients presenting with suggestive clinical features.

## Case presentation

A 15-year-old male presented to the nephrology outpatient department of a tertiary care hospital with complaints of bilateral lower limb swelling, which had developed insidiously over a month and progressively worsened, extending from the foot to the knees. He also reported experiencing facial puffiness, frothing of urine, and shortness of breath for the same duration. Additionally, he disclosed a history of seizure episodes one month prior, for which he had been initiated on anti-epileptic medication.

Upon physical examination, the patient demonstrated orientation to time, place, and person, with noticeable swelling in both lower limbs and facial puffiness. Consequently, he was admitted to the nephrology inpatient department for further evaluation and management. Upon admission, several blood tests were conducted, revealing a low hemoglobin level of 7 g/dL (reference range 11-15 g/dL), elevated white blood cell count of 14,500 cells/mm^3^ (reference range 4,000-11,000 cells/mm^3^), and abnormal kidney function tests. Chest X-ray findings were unremarkable, and electrocardiogram results indicated normal sinus rhythm.

**Figure 1:**
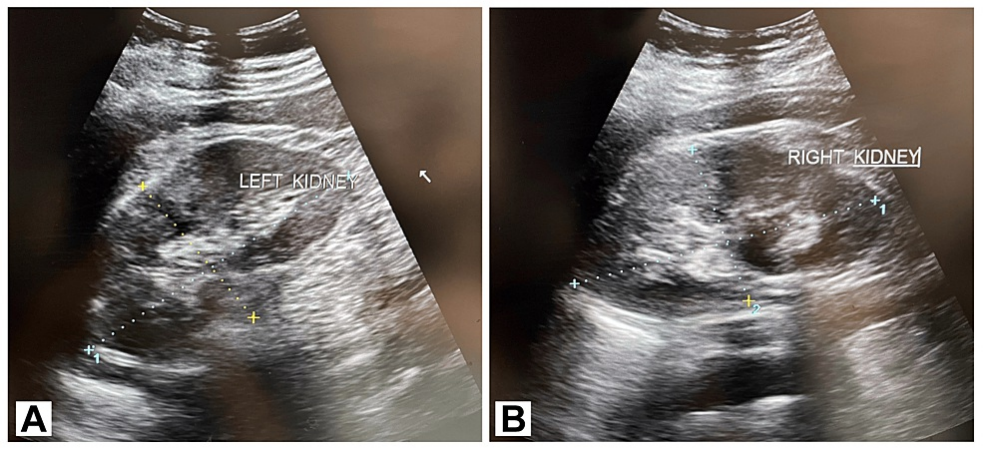
(A) Left kidney measured 11.3 cm x 6.3 cm and (B) right kidney measured about 11.2 cm x 5.8 cm.

Ultrasonography of the abdomen and pelvis revealed edematous bilateral kidneys with altered echotexture and signs suggestive of an infective etiology. The right kidney measured 11.2 cm x 5.8 cm, and the left measured about 11.3 cm x 6.3 cm. Further evaluation included neurological consultations, leading to electroencephalography and magnetic resonance imaging of the brain, which revealed abnormal findings consistent with the patient's clinical presentation (Figures 1A-1B).

**Figure 2:**
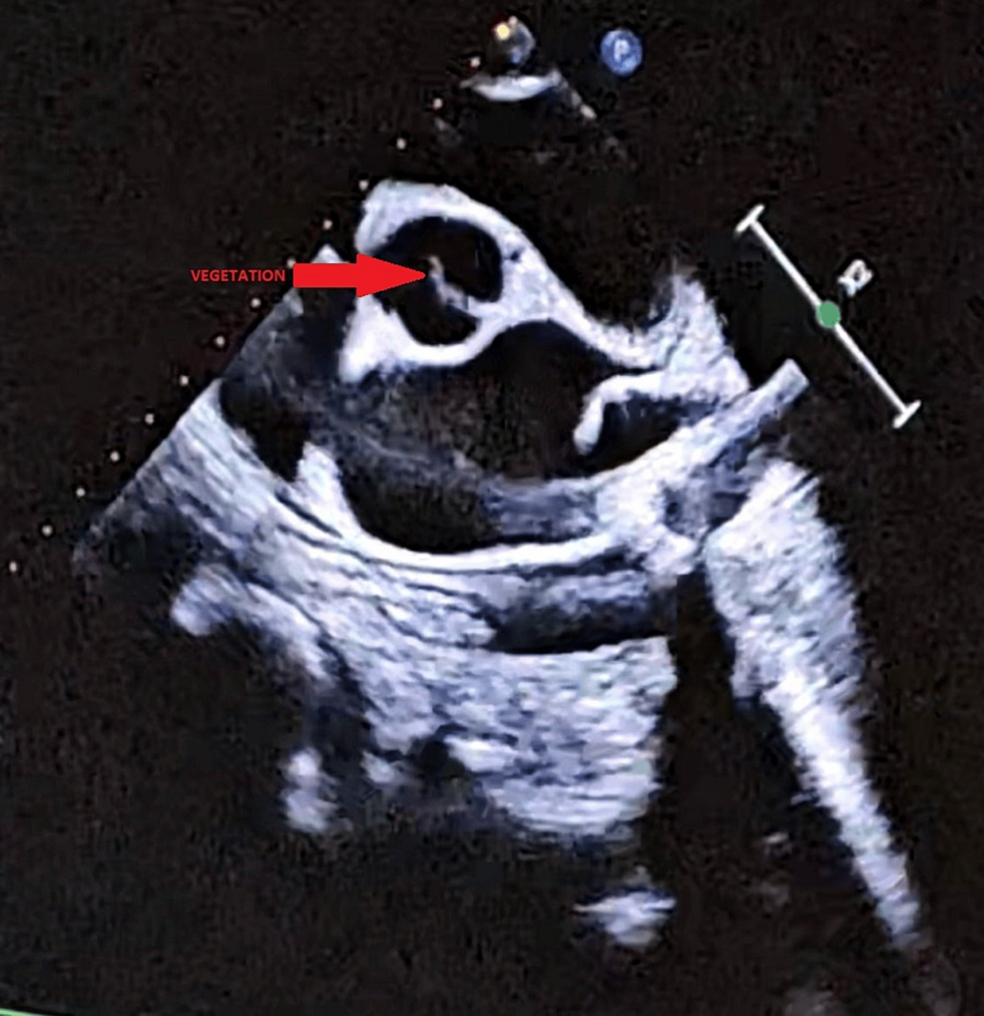
A 2D echocardiogram showing vegetation on the aortic valve leaflet. 2D, two-dimensional

A two-dimensional (2D) echocardiogram demonstrated mild mitral regurgitation, mild tricuspid regurgitation, grade II aortic regurgitation, and vegetation on the aortic valve leaflet, indicative of infective endocarditis (Figure 2).

**Figure 3:**
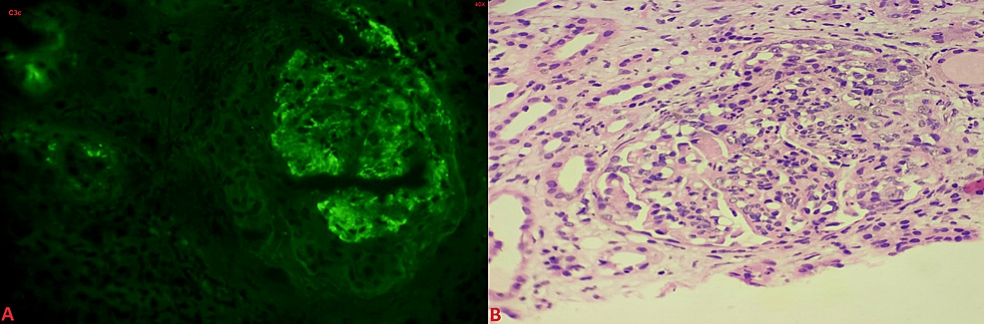
(A) Immunofluorescence microscopy - C3c - 40x - Microphotograph showing peripheral capillary wall and mesangial granular deposits of 2+ to 3+ intensity; (B) H&E (40x) - microphotograph showing a glomerulus with features of proliferative and exudative glomerulonephritis and associated cellular crescent. H&E, hematoxylin and eosin

Subsequently, blood cultures were obtained, yielding growth of Burkholderia cepacia and methicillin-resistant coagulase-negative Staphylococcus. A native kidney biopsy with light microscopy and immunofluorescence was performed, revealing features suggestive of immune complex-mediated crescentic diffuse proliferative and focal exudative glomerulonephritis, raising suspicion for IRGN (Figures 3A-3B).

Following comprehensive clinical, radiological, and pathological assessments, the patient was diagnosed with glomerulonephritis secondary to SBE affecting the aortic valve. Neurological consultation advised continuation of anti-seizure medication. Due to the sudden onset of breathlessness, bilateral coarse crepitations, and tachypnea, the patient was transferred to the medicine intensive care unit, where he received respiratory support via bilevel-positive airway pressure and underwent hemodialysis. Subsequent monitoring and treatment included blood transfusion, diuretic therapy, and a course of antibiotics tailored to the identified pathogens. This case underscores the importance of a multidisciplinary approach in the management of complex medical conditions such as infective endocarditis with renal complications, emphasizing timely diagnosis, supportive care, and targeted therapeutic interventions.

## Discussion

The presented case underscores the importance of recognizing IRGN as a potential SBE complication, particularly in pediatric patients with renal manifestations. IRGN is an immune complex-mediated glomerulonephritis due to bacterial infections, most commonly involving the skin, upper respiratory tract, or heart [2]. The deposition of immune complexes in the glomeruli triggers an inflammatory response, leading to glomerular injury and subsequent renal dysfunction [7]. In the case of SBE, bacteria such as Streptococcus species or Staphylococcus aureus can directly seed the kidney via hematogenous spread, leading to the development of IRGN [8].

The diagnosis of IRGN relies on a combination of clinical features, laboratory findings, and histopathological evaluation. Patients typically present with hematuria, proteinuria, hypertension, and renal insufficiency, often in the setting of an active or recent bacterial infection [9]. Laboratory investigations may reveal elevated inflammatory markers, abnormal kidney function tests, and evidence of systemic inflammation [3]. Kidney biopsy remains the gold standard for confirming the diagnosis of IRGN, with characteristic histopathological findings including glomerular hypercellularity, neutrophil infiltration, and immune complexes on immunofluorescence staining [10].

In the presented case, the patient exhibited classic clinical features of IRGN, including bilateral lower limb swelling, facial puffiness, and frothing of urine in the setting of SBE, confirmed by echocardiogram and blood cultures. The findings of immune complex-mediated glomerulonephritis on kidney biopsy further supported the diagnosis. Management of IRGN secondary to SBE involves addressing both the underlying infection and renal complications. Antibiotic therapy targeted at the causative organism is essential to eradicate the infection and prevent further systemic complications [11]. Supportive measures such as fluid and electrolyte management, blood pressure control, and renal replacement therapy may be necessary to maintain renal function and manage complications such as volume overload and electrolyte imbalances [12]. In severe cases, immunosuppressive therapy may be considered to modulate the inflammatory response and prevent progressive renal damage [13].

This case highlights the importance of a multidisciplinary approach to managing IRGN secondary to SBE, involving collaboration between nephrologists, cardiologists, infectious disease specialists, and intensivists. Timely diagnosis and initiation of appropriate treatment are crucial to optimize outcomes and prevent long-term renal sequelae in affected patients.

## Conclusions

In conclusion, IRGN secondary to SBE is a rare but potentially devastating condition that requires prompt recognition and comprehensive management. This case highlights the importance of a multidisciplinary approach involving nephrologists, cardiologists, infectious disease specialists, and intensivists in diagnosing and treating IRGN. Timely initiation of appropriate antibiotics, supportive care, including respiratory support and renal replacement therapy, and targeted interventions such as kidney biopsy are essential for optimizing outcomes and preventing irreversible renal damage. Further research is warranted to understand better the pathophysiology and optimal management strategies for IRGN secondary to SBE.
